# Contamination and Risk Assessment of Estrogens in Livestock Manure: A Case Study in Jiangsu Province, China

**DOI:** 10.3390/ijerph15010125

**Published:** 2018-01-12

**Authors:** Pengcheng Xu, Xian Zhou, Defu Xu, Yanbing Xiang, Wanting Ling, Mindong Chen

**Affiliations:** 1 Institute of Organic Contaminant Control and Soil Remediation, College of Resources and Environmental Sciences, Nanjing Agricultural University, Nanjing 210095, China; 2015803153@njau.edu.cn (P.X.); 2015103052@njau.edu.cn (X.Z.); 2014103038@njau.edu.cn (Y.X.); 2 Collaborative Innovation Center of Atmospheric Environment and Equipment Technology, Jiangsu Key Laboratory of Atmospheric Environment Monitoring and Pollution Control (AEMPC), Nanjing University of Information Science and Technology, Nanjing 210044, China; defuxu1@163.com

**Keywords:** estradiol equivalent quantity, estrogen, livestock manure, risk assessment, Jiangsu Province, bank up

## Abstract

This study investigated the occurrence and contamination risk of estrogens in livestock manure in Jiangsu Province, China. Four estrogens—estriol (E3), 17β-estradiol (17β-E2), bisphenol A (BPA), and 17α-ethinyloestradiol (EE2)—were detected in livestock manure from hens, ducks, swine, and cows. The respective mean concentrations of each estrogen found in these manures were 289.8, 334.1, 330.3, and 33.7 μg/kg for E3; 38.6, 10.9, 52.9, and 38.8 μg/kg for 17β-E2; 63.6, 48.7, 51.9, and 11.7 μg/kg for BPA; and 14.3, 11.3, 25.1, and 21.8 μg/kg for EE2. Estrogens were most frequently detected at high concentrations in the manure of finishing pigs, followed by the manure of growing pigs and piglets. Estrogens can be partially degraded after banking up for seven days; yet, great quantities of estrogens remain in livestock manure. The total estradiol equivalent quantity (EEQ_t_) estimated to be present in aquatic environments but originating from livestock waste was 10.5 ng/L, which was greater than the hazard baseline value (1 ng/L) and also higher than the proposed lowest observable effect concentration (10 ng/L) of E2 in aquatic environments. The results of our study demonstrate that livestock waste is an important source of estrogens, which may potentially affect the hormonal metabolism of aquatic organisms.

## 1. Introduction

Estrogens, which belong to a group of endocrine-disrupting compounds (EDCs), can be divided into two major groups: natural estrogens, such as estrone (E1), 17α-estradiol (17α-E2), 17β-estradiol (17β-E2), and estriol (E3), and synthetic estrogens, such as bisphenol A (BPA) and 17α-ethinyloestradiol (EE2) [1,2,3]. Their abilities to cause endocrine disruption in non-target species in the environment have been well documented [4,5,6]. Estrogens reach the environment through discharge from sewage treatment plants (STPs) and livestock waste disposal units [7,8]. As early as the 1990s, male fish were found to be feminized in British rivers [9], a phenomenon that was thought to be related to long-term exposure to low concentrations of estrogens in the aquatic environment. This has caused great concern globally. Generally, environmental estrogens have been detected at nanogram per liter levels in many water bodies [10]. However, even at these low levels, the compounds were found to be biologically active [11,12], impacting the reproductive biology of aquatic wildlife (for example, rotifer, shellfish, fish, and batrachian) by disrupting the normal functions of their endocrine systems [13,14]. Furthermore, estrogens are absorbed by the body in a variety of different forms from the environment; this can impair reproductive functions in adults of either sex, lead to irreversible abnormalities when administered during development, and cause cancer [15].

Large quantities of estrogens are present in livestock manure, such as cow dung, swine manure, chicken manure, and duck manure [16,17,18,19,20,21,22]. For instance, Wenzel et al. (1998) observed an estrogen activity (E1 and 17β-E2) of 600 to 1600 μg/kg in cow manure [23]. Raman et al. (2001) measured cow press cake manure and found estrogen concentrations of 32 μg/kg for E1, although levels were undetectable for 17α-E2 and E3; a level of 98 μg/kg was observed for 17β-E2 [19]. Similarly, research by Lange et al. (2002) calculated estrogen excretion for various livestock species [24]. Total estrogens excreted by livestock animals were estimated at about 33 tons/year in the European Union and 49 tons/year in the United States in 2002. Recent work has recognized that biologically significant quantities of estrogens are present in livestock due to hormone excretion [25]. However, little research regarding livestock manure as a source of estrogens in China has been conducted.

Estrogens are excreted into the environment from the wastes of all species and classes of farm animals. Different estrogens are associated with different livestock species [4]. However, data on the excretion of estrogens from different manure types are limited in Asian countries such as China. Furthermore, only limited data are available to address the concentration of estrogens in manure. For instance, Zheng et al. (2007) found that the concentration of estrogens in cow dung is likely to decrease over time [20]. The concentration of estrogens in manure for different livestock animals still needs to be better understood.

China has the largest population of humans and livestock animals in the world, and a portion of livestock farms do not have appropriate facilities for the treatment and disposal of manure [26]. The total excretion of estrogens by humans and animals in China has been estimated at 3069 t/year, with two-thirds of this originating from animals [27]. Therefore, in theory, the animal discharge pathway in China may impose high contamination risks for estrogens in the environment. Estrogens in livestock eventually find their ways into water bodies and/or the soil environment through field manure usage, posing great threats to the ecosystem and human health. As such, actions are wanted to monitor the concentrations of estrogens in livestock manure, and biological and chemical techniques are needed to remove these chemicals from the manure. However, there are few reports available that monitor the estrogen residues in livestock manure in China. To our knowledge, Zhang et al. (2014b, c) measured estrogen concentrations in fresh livestock excreta in East China and in Qi County in China [22,28]. However, data on estrogens in livestock manure are unavailable for other large areas in China, such as Jiangsu Province, which has a relatively developed economic system and a large number of extensive farms.

The major objective of this study was to quantify E3, 17β-E2, BPA, and EE2 concentrations in livestock manure in Jiangsu Province, China. The contamination of estrogens in livestock manure was identified, and the effects of piled-up estrogens in livestock manure were also examined. Additionally, the study attempted to assess the potential risk of estrogens from livestock waste as contaminants of water systems and the potential risks for terrestrial vertebrates, including humans. To our knowledge, this is the first investigation that provides fundamental information about the contamination of estrogens in livestock manure and a risk assessment of the situation in Jiangsu Province, China.

## 2. Materials and Methods 

### 2.1. Chemicals

**Figure 1:**
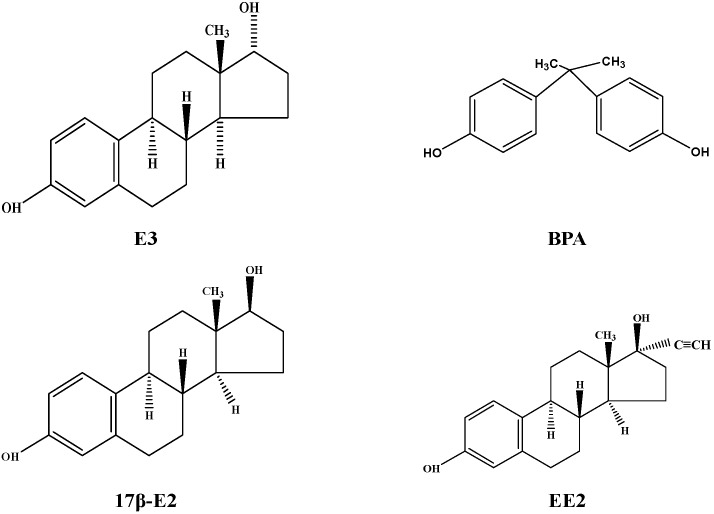
Molecular structures of estriol (E3), 17β-estradiol (17β-E2), bisphenol A (BPA), and 17α-ethinyloestradiol (EE2).

E3, BPA, 17β-E2, and EE2 were purchased from Sigma-Aldrich (≥98%, St. Louis, MO, USA). Individual 10 mg/L chemical stock solutions were prepared in high-performance liquid chromatography (HPLC)-grade acetonitrile and stored at −20 °C. Figure 1 presents the chemical structures of E3, BPA, 17β-E2, and EE2. The physical and chemical properties of these chemicals are shown in Table 1. Methanol and acetonitrile were purchased from TEDIA (Fairfield, OH, USA) and were both graded for HPLC.

### 2.2. Sample Location and Collection Method

Jiangsu is a province located along the eastern coast of China between an east longitude of 116°18′ to 121°57′ and a north latitude of 30°45′ to 35°20′. Jiangsu Province covers an area of 102,600 km^2^. The province has a 954-km-long coastline and a water surface area of 17,300 km^2^. Nanjing is the capital of Jiangsu Province, and has an important geographical position. Samples were collected from the center of Nanjing city and the surrounding area in Jiangsu Province. According to the Animal Husbandry and Veterinary Yearbook [34], breeding-scale animals (cow, swine, and poultry) are larger and, therefore, these animals account for 98.8% of the total area used for animal husbandry. In addition, hens and ducks are the main breeding animals in Jiangsu Province. As a result, we chose to collect the manure of cows, swine, hens, and ducks.

**Figure 2:**
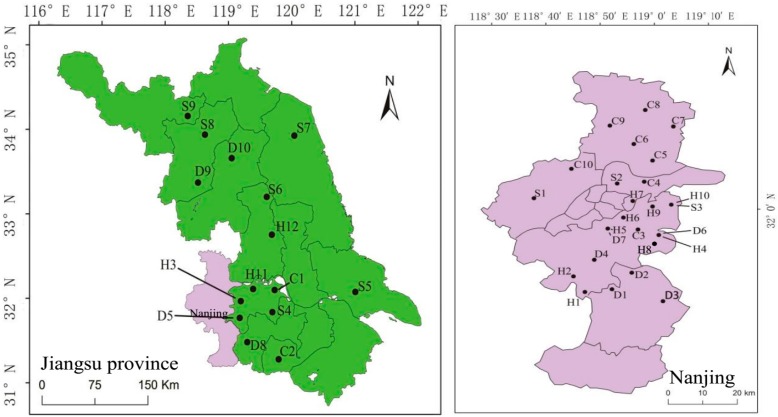
Sampling sites for livestock waste in Jiangsu Province, China. H1–H12 represents 1–12 sampling sites for henneries; D1–D10 represents 1–10 sampling sites for duck farms; C1–C10 represents 1–10 sampling sites for cow farms; and S1–S9 represents 1–9 sampling sites for swine farms.

The livestock manure samples were collected from 12 hen farms, 10 duck farms, 9 pig farms, and 10 dairy farms in October 2015. Figure 2 shows all of the sampling sites. Fresh manure (collected <2 h after deposition) was taken from three typical sampling locations within the farmland barns. At each of these locations, an approximate 500 g sample was collected using an aluminum scoop, and three replicates were conducted. Dry and semisolid samples were placed in plastic bags, transported, and then transferred to plastic bottles upon arrival at the laboratory; wet samples were placed in plastic bottles and transported. Samples were placed in a cooler filled with ice for transport back to the laboratory and then stored at 4 °C until analysis.

### 2.3. Sample Extraction

Freeze-dried samples (1 g) were extracted twice with 15 mL of ethyl acetate. Tubes were closed with a Teflon-liner cap, and the manure was extracted by ultrasonication for 1 h. Samples were centrifuged for 30 min at 4000 r·min^−1^ to separate the manure from the solution. The ultrasound and centrifugation steps were repeated twice. Supernatants were pooled, volatilized with nitrogen, and dissolved in 2 mL methanol, and the volume was brought up to 50 mL with ultrapure water.

The solutions were passed through an activated C_18_-solid phase extraction (SPE) column (200 mg/6 mL) at 3 mL·min^−1^, washed with 5 mL ultrapure water, and pumped for 3 min. A mixture (15 mL) of methanol and ethyl acetate 1:1 (*v*/*v*) was used to elute the samples, and the effluents were collected. All of the samples were filtered through a 0.22 μm organic membrane, before being subjected to HPLC analysis.

### 2.4. Estrogen Detection and Data Analysis

Gas chromatography (GC) coupled with mass spectrometry (MS) (Agilent Technologies Inc., Santa Clara, CA, USA) and liquid chromatography (LC) coupled with MS (Agilent Technologies Inc., Santa Clara, CA, USA) have been developed and used to analyze estrogen levels in different matrixes [21,26,33,35]. Considering the time-consuming nature and high cost of these methods, which make it difficult to analyze a large number of samples, high performance liquid chromatography (HPLC) is also widely accepted as a fast, simple, easy-to-use, and available technique for estrogen analysis in such matrixes [36,37,38,39].

Estrogens were detected using HPLC (LC-20AT; Shimadzu, Kyoto, Japan) with a fluorescence detector (FLD) (RF-10AXL; Shimadzu, Kyoto, Japan) and an Inertsil ODS-SP column (5 μm, 4.6 × 250 mm; GL Sciences, Kyoto, Japan). Methanol/acetonitrile/water (20/30/50, *v*/*v*/*v*) was used as the mobile phase at a flow rate of 0.8 mL/min. HPLC was performed at 40 °C, and the injection volume was 20 μL. The fluorescence excitation and emission wavelengths were 280 nm and 310 nm, respectively. Good linearities with correlation coefficients greater than 0.9995 were observed by the HPLC/FLD detection for the four tested estrogens at 1.00–1000.00 μg/L. The limits of detection of E3, 17β-E2, BPA, and EE2 in manures were 3.354, 5.01, 2.13, and 1.12 μg/kg, respectively. The recoveries of E3, 17β-E2, BPA, and EE2 from different manures (mean ± SD; *n* = 6) were 96.7% ± 10.3%, 92.2% ± 3.98%, 85.9% ± 2.68%, and 86.7% ± 3.34%.

**Figure 3:**
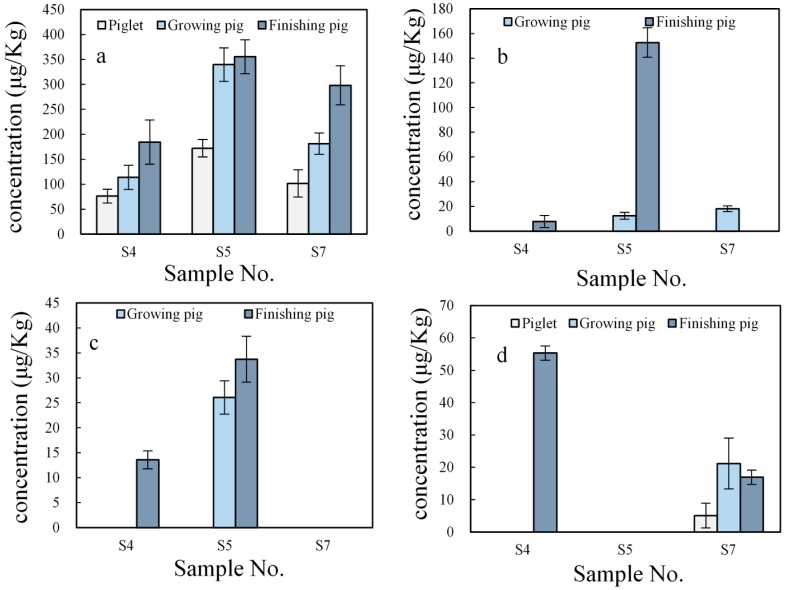
Concentrations of E3 (**a**), 17β-E2 (**b**), BPA (**c**), and EE2 (**d**) in swine at different instars: piglets (body weights less than 20 kg); growing pig (body weights from 25 kg to 45 kg); and finishing pigs (body weights higher than 45 kg). S4, S5, and S7 represent farm numbers.

**Figure 4:**
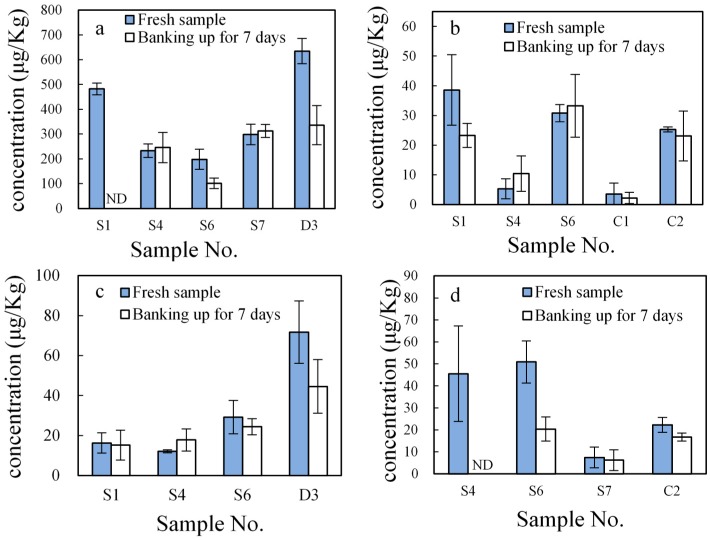
Concentrations of E3 (**a**), 17β-E2 (**b**), BPA (**c**), and EE2 (**d**) in fresh manure and manure that has been banked up for seven days. S1, S4, S6, S7, C1, C2 and D3 represent farm numbers; ND, means under detection limit.

All data were processed using Microsoft Excel 2013 (Microsoft Corporation, Redmond, WA, USA). Each data point in Figure 3 and Figure 4 represents the mean of three replicates, and error bars represent standard deviations (SD).

## 3. Results and Discussion

### 3.1. Occurrence of Estrogens in Livestock Manure

The concentrations of four estrogens in livestock manure are summarized in Table 2 and Table S1. The detection rate (D (%)) of an estrogen in manure was calculated according to the following equation: D (%) = N_detectable_/N_total_ × 100, where N_detectable_ is the number of manure samples with detectable levels of the estrogen under investigation, and N_total_ is the total number of manure samples.

E3, 17β-E2, BPA, and EE2 were detected in hen manure at concentrations of under the detection limit (ND) to 1764.3 (averaged to 289.8), ND to 227.1 (averaged to 38.6), ND to 166.5 (averaged to 63.6), and ND to 67.5 (averaged to 14.3) μg/kg, respectively. The D values in the hen manure were 83.33%, 66.67%, 91.67%, and 58.33%. The concentrations were higher than those found in the manure of broiler chickens reported by Zhang et al. (2014c) (Table 3) [28], which may be because the amount of estrogens in manure varies depending on the sex and age of the poultry [17]. 17β-E2 was documented to range from 14 μg/kg in immature male broilers to 533 μg/kg in hens [40]. The primary estrogen excreted by hens is E3 (Table 2), which is consistent with reports that E1 and E3 are the main estrogens excreted by chickens [41]. As seen in Table 3, 17β-E2 was detected in poultry broiler litter at a concentration of 133 μg/kg [17] and in poultry manure at a concentration of 149.8 μg/kg [21], which are considerably higher than all of the concentrations measured in this study.

As for poultry, previous studies have mainly focused on the estrogens found in hen manure and have seldom reported on the estrogens found in duck manure. However, duck coops are mainly located beside fish farms, which means that estrogens from duck manure are more readily leachable to the aquatic environment. As shown in Table 2, E3, 17β-E2, BPA, and EE2 were detected in duck manure at mean concentrations of 334.1, 10.9, 48.7, and 11.3 μg/kg, with D values of 60%, 70%, 70%, and 50%, respectively. The mean total concentration of the natural estrogens (E3 and 17β-E2) was 345.0 μg/kg in duck manure, which is almost equal to that found in the manure of hens (328.4 μg/kg).

The concentrations of E3, 17β-E2, BPA, and EE2 in swine manure ranged from 174.2 to 518.2, ND to 201.3, ND to 361.8, and ND to 70.1 μg/kg, and their D values were 100%, 66.67%, 66.67%, and 66.67%, respectively. Similarly, average E3 and 17β-E2 concentrations in swine manure were 330.3 and 52.9 μg/kg, which were the highest average concentrations among all of the livestock manure sampled. However, previous studies have reported that free estrogens were detected less frequently or at lower concentrations in swine feces [28]. In addition, swine excrete estrogens mostly in urine [4]. The feces and urine of pigs are excreted in the same place, and it is difficult to clearly separate them. E3 was found in all of the swine manure, which may be due to a mixture of feces and urine.

The mean concentrations of E3, 17β-E2, BPA, and EE2 in cow manure were 33.7, 38.8, 11.7, and 21.8 μg/kg, and their D values were 20%, 80%, 50%, and 50%, respectively. The detection rate for E3 is lowest in cows (20%), and higher among hens (83.33%), ducks (60%), and swine (100%). Zheng et al. (2007) only detected 17α-E2, 17β-E2, and E1 in manure from a dairy farm in California [20], which is consistent with the results of our study. A review by Hanselman et al. also revealed that E3 was not detectable in cow feces, but was detectable in some cow urine [4]. E3 was found at sample sites C5 and C6, which may be due to more regular cleaning. As cleaning is not timed to collect feces, cow dung would be laced with urine, leading to the existence of E3 in cow manure. The concentration of 17β-E2 was ND–88.3 μg/kg in cow manure. Zhang et al. (2014c) measured 17β-E2 concentrations ranging from 75.2 to 98.2 μg/kg in fresh cow manure [28], which is almost within the same order of magnitude as the concentrations measured in our study.

As for the synthetic estrogens (BPA and EE2), the mean concentrations of BPA found in the manure of hens, ducks, swine, and cows were 63.6, 48.7, 51.9, and 11.7 μg/kg, respectively. Except for the cow manure, BPA was found at similar concentration levels in the manure of the different animal farms. BPA has also been found at levels between 61.1 and 1112 μg/kg (dry weight) in the ten liquid manure samples analyzed in a previous study [1]. Similarly, BPA has been found in raw swine wastewater, and feed items or other equipment used in swine farms could have been a possible source [35]. Another possible source for the high levels of BPA present in the manure samples could be migration from the materials used to coat the inner surface of the animals’ food tanks [1]. Zhang et al. (2014b) assumed that BPA and EE2 were continually excreted in feces and urine, and the daily excretions of BPA and EE2 were up to 43.99 μg/day/cow and 82.69 μg/day/cow, respectively [22]. EE2, as an orally bio-active estrogen, is one of the most commonly used medications for humans as well as livestock (including those used in aquaculture activity) [3]. In many countries, the oral contraceptive pill including EE2 is frequently used as a form of birth control [46]. EE2 is also utilized to improve productivity by promoting growth, and to prevent and treat reproductive disorders in livestock [3,47,48,49]. This compound was found to have a mean concentration of 17.8 μg/kg in all manure samples, suggesting that EE2 has been used to increase the body weight of animals or treat reproductive disorders in some animal farms.

### 3.2. Impact of Livestock Growth Stages on Estrogen Concentrations in Manure

Different estrogens are associated with livestock at different stages of growth [4]. In swine, it has been suggested that the concentration of estrogens are higher in sow waste than in barrow waste [28]. The estrogen concentrations measured in swine manure from sites S4, S5, and S7 varied extensively with growth stages, as shown in Figure 3. Other studies have also considered the relationship between livestock growth stages and estrogen concentrations in manure [50]. Regardless of the livestock growth stage, E3 was detected in all of the samples from the three swine farms, and the concentration of E3 was always significantly higher than that of other estrogens. In addition, with increasing swine instar, the concentration of E3 rises dramatically. 17β-E2 and BPA were not detectable in the manure of piglets. In the samples from site S7, 17β-E2 was the main estrogen excreted by swine [25]. It was also found in the manure of growing pigs, but was undetectable in the manure of piglets and finishing pigs. This phenomenon is probably attributable to the diet or health status of the animals, which contributes to excretion rates [4]. The most interesting finding was that estrogens were most frequently detected with highest concentrations in the manure of finishing pigs, followed by the manure of growing pigs and piglets. This confirms that the estrogen content is rising with increasing swine instar.

### 3.3. Impacts of Banking Up Estrogen Concentrations in Pig Manure

After being collected from the barn, manure is usually banked up in the short term. Other measures to treat the manure, such as transport and fertilization, have been widely adopted. Zheng et al. (2007) found that the concentration of estrogens in cow dung will decrease after a period of time being piled up [20]. However, little data is available that addresses the concentration of estrogens in manure from different animals after being piled up.

Figure 4 shows the estrogen concentrations in manure from sites S1, S4, S6, S7, C1, C2, and S7 (as representatives), which were banked up for seven days. The concentrations of EE2 in the livestock manure samples from S4, S6, S7, and C2 decreased by 100%, 60.07%, 15.90%, and 24.81%, respectively, after a 7 day pile up. This may have been caused by microbial biodegradation and/or photodegradation [51,52]. Yoshimoto et al. (2004) identified microorganisms with a great ability to degrade E3, and, as a result, E3 at initial concentrations of 100 mg/L was dramatically reduced by microbial biodegradation [53]. In addition, previous studies have reported that estrogen can be degraded under light that has a wavelength of 290–720 nm [54]. Here, the concentrations of E3 in S1, S6, and D3 were significantly lower after a 7 day pile up. Nevertheless, few significant changes were observed for E3 concentrations in samples from S4 and S7 after 7 day treatments. Several studies have shown that microorganisms are able to partially transform E1 into E2 and produce other more polar intermediate compounds such as E3 [25,31]. In some manure samples (e.g., S6 and D3 for BPA and S1 and C1 for 17β-E2), the concentrations of BPA and 17β-E2 were lower; however, in other samples, the concentrations changed little or had increased after banking up for 7 days (Figure 4). Khanal et al. (2006) showed that conjugated estrogens could be transformed into free estrogens by hydrolysis under specified conditions [2], which may be a reason for the increase of estrogens in livestock manure after banking up for 7 days.

Notably, the concentrations of our four test estrogens in livestock manure may have decreased due to partial chemical and/or biological degradation after banking up. However, they did not completely degrade or disappear; rather, they remained at significant concentration levels in treated manure, which poses a potential risk in terms of environmental contamination.

### 3.4. Potential Risk of Estrogens in Livestock Manure

Recently, the adverse effects of estrogens (including both natural and synthesized compounds) in water environments have been widely reported. However, the impact of livestock waste as a source of estrogens in aquatic environments is still being uncovered. Given the strong links between estrogen water content and endocrine disruption in fish, it is necessary to assess the quantities and identify the sources of estrogens that are entering the aquatic environment. Livestock waste containing estrogens has entered the waterways through manure runoff [55]. Based on estimated quantities of estrogens in municipal wastewater treatment plants and annual runoff, Johnson et al. (2006) estimated the concentration of estrogens in Japanese and British rivers [56]. Similarly, Liu et al. (2013) calculated the concentrations of estrogens in livestock waste and the potential risk in three provinces in Northeast China [57]. In this study, models were used to estimate water concentrations of estrogens derived from livestock waste in Jiangsu Province.

The amount of livestock manure present on a farm is related to the population of animals, the species, the feeding cycles, and the excretion of waste coefficient over one year. Animal census figures were complete up to 2014, which included information on cows, swine, and poultry from the Animal Husbandry and Veterinary Yearbook (AHV, 2015) [34]. However, the estimated amount of duck manure is excluded due to an absence of data for these animals. Given the occurrence of estrogens found in livestock manure in this study, not only does the concentration of natural estrogen in duck manure equal that in the manure of hens, but both chickens and ducks belong to the same subspecies of poultry. Therefore, our assessment suggests that hens be regarded as laying poultry and ducks be regarded as table poultry. Table 4 summarizes the quantity of livestock waste in 2014 in Jiangsu Province. Jiangsu Province is located in a subtropical zone and a warm temperate zone; farm animals include cows, swine, table poultry, and laying poultry, and these species account for 98.8% of the total number of farm animals (the proportion of other animals like sheep, camels, horses, and rabbits is negligible). As such, it may be reasonable to expect that sheep, camels, horses, and rabbits contribute little to the load of estrogens in the environment.

The excretion coefficient of waste discharged from livestock is derived from the amount of waste produced by an individual animal per day, which is related to the population, growth stage, sex, and feeding pattern of the animal. For this study, and in combination with Jiangsu provincial data, excretion coefficients of waste discharged from livestock are listed in Table 4. Excretion coefficients of manure waste have been identified by experimentation [58]. Combining feeding cycle information (Table 4) [58], the quantity of livestock waste can be calculated using Equation (1):

q_i_ = m_i_ × d_i_ × p_i_ (1)

where q_i_ is the quantity of livestock waste (10^6^ kg), m_i_ is the amount of livestock on hand (10^6^ kg), d_i_ is the feeding cycle of the livestock (days), and p_i_ is the excretive coefficient obtained from the livestock per day (kg/day). As to the quantity of estrogens in livestock, our approach can be summarized in Equations (2) and (3):

QEM = QLM × (1 − WC) × MV(2)

where QEM is the quantity of estrogens (kg) in livestock manure in 2014 in Jiangsu Province, China (Table 5); QLM is the quantity of livestock manure (10^6^ kg); WC is the water content of the livestock manure [59], which is essential for measurable manure samples; and MV is the mean value of estrogen (μg/kg); and

QEU = QLU × MV(3)

where QEU is the quantity of estrogens (kg) in livestock urine in 2014 in Jiangsu Province, China (Table 6) and QLU is the quantity of livestock urine (10^6^ kg). MV is the mean value of estrogen in livestock urine (μg/kg) [28]. Combining QEM and QEU with Equations (2) and (3), predictions for total QEM and QEU are shown in Table 5 and Table 6.

It is difficult to predict how much estrogen would escape soil attenuation and reach adjacent river systems in Jiangsu Province. However, a great deal of literature exists on losses from soil nutrient applications, which may help provide a reference point. Previous studies have reported that nutrients in runoff and water losses are up to 30–40% [55]. Considering that the study area belongs to the Yangtze River Basin in China, this implies that losses of estrogens from livestock waste in the soil are also approximately 30%. Predicted environmental concentrations of estrogen in ground water and surface water are calculated using Equation (4):

PEC_i_ = (E_i_ × η) ÷ r (4)

where PEC_i_ is the predicted environmental concentration of estrogens in the ground water and surface water in Jiangsu Province (ng/L), E_i_ is QEM or QEU (kg/a), η is the loss rate of estrogens from livestock waste in Jiangsu Province (η = 30%), and *r* is the quantity of ground water and surface water in 2004 in Jiangsu Province (r = 3.9934 × 10^13^ L/a) (NBSPRC, 2014 [60]).

To confirm the ecological and environmental risks of estrogens discharged from livestock waste, previous studies have agreed that estradiol equivalent (EEQ) values are evaluated with an E2 equivalent factor (EEF) for estrogenic chemicals. The EEQs of E3, BPA, 17β-E2, and EE2 were calculated according to Equation (5), and the total estradiol equivalent quantity was calculated according to Equation (6):

EEQ_i_ = EEF_i_ × PEC_i_, (5)

(6) $${\mathrm{EEQ}}_{t}=\sum_{i=1}^{n}{\mathrm{EEQ}}_{i}$$

where EEQ_i_ is the estradiol equivalent quantity of estrogen (ng/L); EEF_i_ is the estradiol equivalency factor of estrogen; 17β-E2 = 1, E3 = 0.054, BPA = 0.00011, and EE2 = 10 [9,61]; PEC_i_ is the predicted environmental concentration of estrogen (ng/L); and EEQ_t_ is the total estradiol equivalent quantity (ng/L).

The EEQ_t_ is used to evaluate the potential risk of estrogens in livestock waste; this is shown in Table 7. The European Commission has declared the concentration causing endocrine-disrupting effects to be 1 ng/L, indicating that substances with EEQ larger than 1 ng 17β-E2/L are likely to affect the endocrine systems of aquatic organisms exposed to the contaminated water (European Commission 1996 [62]). EEQ_t_ in our study amounted to 10.5 ng/L, which is greater than the hazard baseline value of 1 ng/L and also higher than the proposed lowest observable effect concentration of E2 (10 ng/L) (European Commission 1996 [62]). This clearly indicates that estrogens derived from livestock manure in Jiangsu Province may be particularly harmful to aquatic organisms.

It should be noted that the calculation of the EEQ_t_ concentration of estrogens contributed by livestock waste did not consider dilution with water, degradation and half-life potential, adsorption, or biodegradation of estrogens in an aqueous environment. In addition, only four estrogens were investigated; there may be other estrogens (e.g., 17α-estradiol [21,33,44], estrone [22,28,33,44], equol [35]) that exist in livestock manure that pose contamination risks. However, our primary investigation showed that livestock waste is an important source of estrogens and that this may potentially affect the hormonal metabolism of aquatic organisms.

The results of this study tell us that the monitoring of estrogens in the aquatic environment is necessary to confirm the potential risk and ensure the safety of drinking water, and continued research is needed to examine other potent estrogens with harmful properties in livestock manure. Moreover, monitoring should be expanded to include conjugates, sludge-bound estrogens, and other estrogen agonists and antagonists. On the other hand, further treatment of livestock waste is necessary to remove the estrogens from livestock manure. Recently, a biological technique was proposed for the removal of estrone, 17β-estradiol, and estriol from cow manure by immobilized degrading-strain *Novosphingobium* sp. ARI-1 [63]. A Fenton oxidation method was investigated to simultaneously remove different estrogens including estriol, bisphenol A, diethylstilbestrol, estradiol, and ethinyl estradiol from cow manure [64]. However, more cost-effective and available techniques are still wanted in future.

## 4. Conclusions

Our study investigated the occurrence and contamination risk of estrogens in livestock manure in Jiangsu Province, China. Four estrogens (E3, 17β-E2, BPA, and EE2) were detected at high concentrations in the livestock manure of hens, ducks, swine, and cows. Estrogen content in manure generally rose with increasing swine instar, and could be partially degraded after banking up for seven days; yet, great amounts of estrogens remained in the livestock manure. The estimated EEQ_t_ (10.5 ng/L) of the aquatic environment contributed by livestock waste was greater than the hazard baseline value and was also higher than the proposed lowest observable effect concentration of E2 in aquatic environments. The results of this investigation show that livestock waste is an important source of estrogens, which may potentially affect the hormonal metabolism of aquatic organisms.

## Figures and Tables

**Table 1:** General physicochemical properties of estrogens used in this study.

Parameters	E3	BPA	17β-E2	EE2	Reference
MW (g/mol)	288.4	228.3	272.0	296.4	[29,30]
VP (Pa)	6.7 × 10^−5^	4.0 × 10^−8^	2.3 × 10^−3^	4.5 × 10^−11^	[29,30,31,32]
pK_a_	10.4	10.7	10.5	11.3	[30,33]
logK_ow_	2.60	3.30	4.15	3.64	[29]
S_w_ (mg/L)	13	12	13	4.8	[29,30]

MW = molecular weight. VP = vapor pressure. K_a_ = acid ionization constant. K_ow_ = octanol–water partition coefficient. S_w_ = solubility in water. E3 = estriol. 17β-E2 = 17β-estradiol. BPA = bisphenol A. EE2 = 17α-ethinyloestradiol.

**Table 2:** Concentrations of estrogens in manures of hen, duck, swine and cow.

Type		E3 (μg/kg)	17β-E2 (μg/kg)	BPA (μg/kg)	EE2 (μg/kg)
Hen (*n* = 12)	Concentration	ND–1764.3	ND–227.1	ND–166.5	ND–67.5
	Mean	289.8	38.6	63.6	14.3
	Detection rate	83.33%	66.67%	91.67%	58.33%
Duck (*n* = 10)	Concentration	ND–1155.4	ND–45.6	ND–178.9	ND–43.4
	Mean	334.1	10.9	48.7	11.3
	Detection rate	60%	70%	70%	50%
Swine (*n* = 9)	Concentration	174.2–518.2	ND–201.3	ND–361.8	ND–70.1
	Mean	330.3	52.9	51.9	25.1
	Detection rate	100%	66.67%	66.67%	66.67%
Cow (*n* = 10)	Concentration	ND–240.9	ND–88.3	ND–33.3	ND–106.3
	Mean	33.7	38.8	11.7	21.8
	Detection rate	20%	80%	50%	50%
Total (*n* = 41)	Concentration	ND–1764.3	ND–227.1	ND–361.8	ND–106.3
	Mean	247.0	35.0	44.7	17.8
	Detection rate	65.85%	70.73%	73.17%	56.10%

The detection rate (D (%)) of an estrogen in manure was calculated according to the following equation: D (%) = N_detectable_/N_total_ × 100, where N_detectable_ is the number of manure samples with detectable levels of the estrogen under investigation, and N_total_ is the total number of manure samples. ND = under detection limit. E3 = estriol. 17β-E2 = 17β-estradiol. BPA = bisphenol A. EE2 = 17α-ethinyloestradiol.

**Table 3:** Documented concentrations of estrogens in livestock manures.

Location	Livestock Manure	Estrogens Concentration (μg/kg)				Reference
		E1	αE2	βE2	E3	
The Netherlands	Calves feces	NM	NM	2.3	NM	[16]
	Manure	28–72	120–190	46–50	NM	[42]
The United States	Poultry broiler litter	NM	NM	133	NM	[17]
	Poultry broiler litter	NM	NM	20–35	NM	[18]
	Cow press cake manure	32	ND	98	ND	[19]
	Swine finishing hoops	217	150	ND	NM	[4]
	Swine farrowing pit	4728	890	1215	NM	
	Dairy dry-stack semisolid ^a^	300	500	160	NM	[8]
	Dairy dry-stack solid ^a^	180	<100	180	NM	
	Swine finisher hoop structure ^a^	300	270	<100	NM	
	Fresh dairy manure	535	1416	153	ND	[20]
	Piled dairy manure (2 weeks age)	697	172	37	NM	
	Poultry manure	44.2	92.7	149.8	NM	[21]
	Cow manure	16.1	6.2	16.6	NM	
	Poultry manure	54.15	2.68	4.98	8.13	[43]
France	Swine manure ^b^	ND–3282	ND–1594	ND–343	149–3259	[44]
	Suspended solid swine manure	6–1209	4–139	2–181	ND–315	
Vietnam	Fresh cow manure	NM	NM	3.63	NM	[45]
China	Milking cow feces	ND–9.7	NM	21.8–101.0	ND	[22]
	Piglet feces	ND	NM	17.9–22.2	ND	
	Barrow feces	ND–1.9	NM	ND–3.7	ND	
	Sow feces	ND	NM	ND	ND	
	Broiler (female) feces	9.4–12.8	NM	52.2–60.2	11.8–13.1	
	Broiler (male) feces	ND	NM	ND	ND	
	Laying hen feces	2.3–27	NM	22.6–73	ND–12.9	
	Brood hen feces	ND	NM	ND	ND	
	Milking cow feces	26.8–38.2	NM	75.2–98.2	ND	[28]
	Beef cattle feces	ND	NM	17.3–25.9	ND	
	Sow feces	5.6–6.2	NM	ND	ND	
	Broiler chicken	21.1–22.1	NM	11.3–15.7	12.9–14.5	

NM = not measured. ND = under detection limit. E1 = estrone. 17α-E2 = 17α-estradiol. E3 = estriol. 17β-E2 = 17β-estradiol. ^a^ Estimated from the graph provided in the reference; ^b^ Data for liquid sample (units ng/L).

**Table 4:** The quantity of livestock wastes in 2014 in Jiangsu Province, China.

Type	Cow	Swine	Table Poultry	Laying Hen	Others
Amount of livestock in hand (10^6^) ^a^	0.204	17.873	158.4475	158.4475	4.031
Excretive coefficient of faces (kg/day) ^b^	30	2.2	0.15	0.075	-
Excretion coefficient of urine (kg/day) ^b^	18	2.9	-	-	-
The feeding cycle (days)	365	180	55	210	-
The quantity of livestock manure (10^6^ kg)	2233.80	7077.708	13,071.9019	2495.5481	-
The quantity of livestock urine (10^6^ kg)	1340.28	9329.706	-	-	-

^a^ AHV (2015) [34]; ^b^ Liu et al. (2002) [58], means not available.

**Table 5:** The quantity of estrogens in livestock manure in 2014 in Jiangsu Province, China.

Type			E3		BPA		17β-E2		EE2	
Species	QLM (10^6^ kg)	WC	MV (μg/kg)	QEM (kg)	MV (μg/kg)	QEM (kg)	MV(μg/kg)	QEM (kg)	MV (μg/kg)	QEM (kg)
Cow	2233.80	85%	33.7	11.29	11.7	3.92	38.8	13.00	21.8	7.30
Swine	7077.71	73%	330.3	631.20	51.9	99.18	52.9	101.09	25.1	47.97
Table poultry	13,071.90	75%	334.1	1091.83	48.7	159.15	10.9	35.62	11.3	36.93
Laying hen	2495.55	75%	289.8	180.80	63.6	39.68	38.6	24.08	14.3	8.92
Total				1915.12		301.93		173.79		101.12

E3 = estriol. 17β-E2 = 17β-estradiol. BPA = bisphenol A. EE2 = 17α-ethinyloestradiol. QLM is the quantity of livestock manure (10^6^ kg). MV is the mean value of estrogen (μg/kg). QEM is the quantity of estrogens in livestock manure in 2014 in the Jiangsu Province, China (kg). WC is the water content of livestock manure.

**Table 6:** The quantity of estrogens in livestock urine in 2014 in Jiangsu Province, China.

Type		E3		BPA		17β-E2		EE2	
species	QLU (10^6^ kg)	MV (μg/kg)	QEU (kg)	MV (μg/kg)	QEU (kg)	MV (μg/kg)	QEU (kg)	MV (μg/kg)	QEU(kg)
cow	13.4028	ND	-	383	513.33	ND	-	ND	-
swine	93.29706	204	1903.26	380	3545.29	ND	-	ND	-
Total			1903.26		4058.62				

E3 = estriol. 17β-E2 = 17β-estradiol. BPA = bisphenol A. EE2 = 17α-ethinyloestradiol. QLU is the quantity of livestock urine (10^6^ kg). MV is the mean value of estrogen (μg/kg). QEU is the quantity of estrogens in livestock urine in 2014 in the Jiangsu Province. ND = under detection limit. - means not available.

**Table 7:** The predicted environment concentration of estrogens in the water in Jiangsu Province.

Parameters	E3	BPA	17β-E2	EE2
QEM (10^12^ ng)	1915.12	301.93	173.79	101.12
QEU (10^12^ ng)	1903.26	4058.62	-	-
PEC_i_ (ng/L)	28.69	32.76	1.31	0.76
EEQ_i_ (ng/L)	1.549	0.004	1.31	7.6
EEQ_t_ (ng/L)	10.5			

E3 = estriol. 17β-E2 = 17β-estradiol. BPA = bisphenol A. EE2 = 17α-ethinyloestradiol. - means not available. QEU is the quantity of estrogens in livestock urine in 2014 in the Jiangsu Province. PEC_i_ is the predicted environment concentration of estrogen i in the ground water and surface water in the Jiangsu Province (ng/L). EEQ_i_ is the estradiol equivalent quantity of estrogen i (ng/L). EEQ_t_ is the total estradiol equivalent quantity (ng/L). QEM is the quantity of estrogens in livestock manure in 2014 in the Jiangsu Province, China (kg).

## Supplementary Materials

The following are available online at www.mdpi.com/1660-4601/15/1/125/s1, Table S1: The basic data of estrogens in livestock manure samples.
