# Supplementary material for: Contamination and Risk Assessment of Estrogens in Livestock Manure: A Case Study in Jiangsu Province, China
Source: Int J Environ Res Public Health. 2018 Jan 12;15(1):125. doi: 10.3390/ijerph15010125 (PMC5800224; doi:10.3390/ijerph15010125)
Supplement: Supplementary file 1 [file ijerph-15-00125-s001.pdf]

Table S1. The basic dates of estrogens in livestock manure samples.

Sample id	E3 (μg/kg)	17β-E2 (μg/kg)	BPA (μg/kg)	EE2 (μg/kg)
H1	207.1 ± 33.7	ND	47.2 ± 8.7	13.4 ± 1.5
H2	309.0 ± 14.5	227.1 ± 23.4	23.9 ± 3.9	ND
H3	87.5 ± 9.1	106.8 ± 11.1	149.2 ± 39.2	57.5 ± 9.5
H4	86.6 ± 7.6	57.8 ± 4.6	95.4 ± 13.7	67.5 ± 19.3
H5	ND	17.8 ± 11.1	ND	ND
H6	3.6 ± 2.6	24.2 ± 3.0	23.6 ± 1.6	ND
H7	ND	11.7 ± 2.6	74.1 ± 10.1	3.3 ± 1.2
H8	1764.3 ± 139.8	ND	111.5 ± 60.0	ND
H9	23.5 ± 7.0	3.5 ± 0.5	5.4 ± 2.9	ND
H10	180.4 ± 32.6	14.6 ± 1.4	12.7 ± 7.8	7.2 ± 4.1
H11	645.2 ± 114.4	ND	53.6 ± 8.8	14.0 ± 4.6
H12	170.9 ± 9.7	ND	166.5 ± 24.4	8.6 ± 8.6
D1	1155.4 ± 33.2	20.1 ± 5.0	55.0 ± 9.8	28.6 ± 3.5
D2	ND	17.9 ± 5.6	ND	2.7 ± 1.6
D3	634.6 ± 310.8	ND	71.7 ± 15.6	ND
D4	881.3 ± 203.3	2.8 ± 1.8	23.7 ± 2.3	ND
D5	139.2 ± 51.4	ND	ND	32.0 ± 3.8
D6	ND	9.5 ± 1.3	178.9 ± 38.7	ND
D7	418.6 ± 213.8	ND	ND	6.2 ± 3.7
D8	ND	45.6 ± 8.9	49.3 ± 15.7	ND
D9	ND	8.4 ± 2.8	25.2 ± 2.8	ND
D10	112.2 ± 20.9	4.3 ± 0.9	82.8 ± 24.0	43.4 ± 8.4

S1	518.2 ± 23.6	38.6 ± 11.9	16.3 ± 5.1	ND
S2	514.5 ± 76.9	ND	13.8 ± 1.4	8.0 ± 2.3
S3	197.8 ± 26.9	47.4 ± 28.0	ND	70.1 ± 33.0
S4	174.2 ± 27.3	5.2 ± 3.4	12.1 ± 0.7	45.5 ± 21.7
S5	186.4 ± 41.6	152.6 ± 11.9	33.7 ± 8.6	ND
S6	197.9 ± 40.7	30.8 ± 2.9	29.2 ± 8.9	50.9 ± 19.6
S7	298.2 ± 5.9	ND	ND	7.4 ± 4.7
S8	460.2 ± 17.9	ND	ND	ND
S9	425.4 ± 52.4	201.3 ± 22.4	361.8 ± 39.1	43.9 ± 26.7
C1	ND	2.2 ± 1.9	ND	ND
C2	ND	30.5 ± 1.0	ND	22.2 ± 3.4
C3	ND	86.7 ± 5.0	ND	44.3 ± 2.8
C4	ND	73.9 ± 9.8	ND	ND
C5	95.8 ± 8.9	44.9 ± 10.0	33.3 ± 4.5	11.4 ± 2.4
C6	240.9 ± 70.3	ND	22.3 ± 1.1	ND
C7	ND	ND	19.5 ± 6.4	ND
C8	ND	42.0 ± 3.9	31.9 ± 8.4	ND
C9	ND	19.8 ± 1.1	9.6 ± 1.1	33.6 ± 7.1
C10	ND	88.3 ± 27.9	ND	106.3 ± 5.3

ND means under detection limit. H1–12 represents 1–12 sampling sites for henneries; D1–10 represents 1–10 sampling sites for duck farms; C1–10 represents 1–10 sampling sites for cow farms; and S1–9 represents 1–9 sampling sites for swine farms. E3: estriol, 17β-E2: 17β-estradiol, BPA: bisphenol A, EE2: 17α-ethinyloestradiol.
